# Effect of HIV infection-related factors on SVR rate in HCV treatment in HIV-infected patients

**DOI:** 10.7448/IAS.17.4.19635

**Published:** 2014-11-02

**Authors:** Alexey Kravchenko, Uliana Kuimova

**Affiliations:** Central Scientific Research Institute of Epidemiology, Russia AIDS Federal Centre, Moscow, Russian Federation

## Abstract

**Introduction:**

Factors that have an effect on the rate of sustained virological response (SVR) in chronic hepatitis C (CHC) patients include: genotype of hepatitis C virus (HCV); level of HCV RNA replication and rate of its reduction in the course of treatment; original hepatic fibrosis level; genotype of Interleukin-28B (especially for Genotype 1 HCV – G1); daily ribavirin (RBV) dose. This study evaluated the effect of the HIV infection-related factors on the SVR rate in HCV treatment in patients with concurrent infection (HIV/HCV).

**Methods:**

The follow-up included 232 HIV/HCV-infected patients. Ninety-nine of 232 patients with HIV/HCV-infection received antiretroviral therapy (ART) for at least three months before the initiation of the CHC treatment. Before the HCV therapy, the median of CD4+cells was 406/mm^3^ (with ART) and 507/mm^3^ (without ART). Patients received HCV treatment with pegylated interferon (PEG-IFN) and RBV (1000/12,000 mg/day) during 24–48 weeks.

**Results:**

SVR was received in 50% of patients with G1 HCV, and 80.1% of patients with Genotypes 2/3 (G2/3; p<0.0001). The SVR rate in the group of patients without ART was reliably higher, 74.4% (with ART – 58.6%; p=0.0053). No significant differences in the SVR rate (62.3% and 69.6%, accordingly) were detected after the differentiation of patients based on the initial absolute values of CD4+cells count (<350 cells/mm^3^ and >350 cells/mm^3^). In 127 patients with the HIV/HCV-infection, the percentage of CD4+cells before the CHC treatment was >25% and more (Group 1 [Gr. 1]), and in 105 patients ≤25% (Group 2 [Gr. 2]). The SVR rate for Gr. 1 patient was 74.6%, and for Gr. 2 patients –58.1% (p=0.0023). The SVR rate in patients with G1 HCV was 56.8% (Gr. 1) and 44.2% (Gr. 2; p=0.1095), whereas the rate for G2 and 3 was 85.5% and 71.7%, accordingly (p=0.0242). Forty patients in Gr. 1 and 59 patients in Gr. 2 received ART. The comparison of the SVR rate for these patients showed no significant differences: 60% and 57.6%, accordingly. SVR rate in the patients without ART demonstrated that for Gr.1 patients (CD4+>25%) was reliably higher, 82.8% (compared to Gr.2 with 58.7%; p=0.0012).

**Conclusions:**

Along with factors related to HCV and the patient, the SVR rate in the HCV treatment with PEG-IFN and RBV may be affected in patients with the concurrent infection by the use of ART and original relative content of CD4+cells. The maximum SVR rate was achieved in the patients without ART and with the CD4+cells >25% (baseline). When indicted, it is reasonable to provide HCV treatment to HIV-infected patients as long as the percentage of CD4+cells remains high and there is no need of ART.

